# Transition to diversification by competition for multiple resources in catalytic reaction networks

**DOI:** 10.1186/s13322-015-0010-1

**Published:** 2015-04-02

**Authors:** Atsushi Kamimura, Kunihiko Kaneko

**Affiliations:** Department of Basic Science, The University of Tokyo, 3-8-1, Komaba, Meguro-ku, 153-8902 Tokyo Japan

**Keywords:** Protocells, Resource competition, Catalytic network

## Abstract

**Background:**

All life, including cells and artificial protocells, must integrate diverse molecules into a single unit in order to reproduce. Despite expected pressure to evolve a simple system with the fastest replication speed, the mechanism by which the use of a great variety of components, and the coexistence of diverse cell-types with different compositions are achieved is as yet unknown.

**Results:**

Here we show that coexistence of such diverse compositions and cell-types is the result of competitions for a variety of limited resources. We find that a transition to diversity occurs both in chemical compositions and in protocell types, as the resource supply is decreased, when the maximum inflow and consumption of resources are balanced.

**Conclusions:**

Our results indicate that a simple physical principle of competition for a variety of limiting resources can be a strong driving force to diversify intracellular dynamics of a catalytic reaction network and to develop diverse protocell types in a primitive stage of life.

**Electronic supplementary material:**

The online version of this article (doi:10.1186/s13322-015-0010-1) contains supplementary material, which is available to authorized users.

## Background

Cells, even in their primitive forms [1-3], must integrate diverse molecules into a single unit so that they keep reproduction where they sustain similar chemical compositions. In main scenarios for the origin of life, through the path from a mixture of organic component (the primeval soup with diverse molecular species) to reproducing cells, the emergence of replicating entities has been required for homeostatic growth capable of undergoing Darwinian evolution [4-7].

For faithful replications, the hypercycle model in which different molecular species mutually catalyze the replication of each other [8,9] provides a basic mechanism to overcome an inevitable loss in catalytic activities through mutations. Protocells encapsulating such hypercycles can exhibit robust reproduction even in the presence of parasitic molecules that may destroy the mutual catalytic reactions [10-16].

Despite recent recognition of protocells as a stepping-stone to the origin of life [1,17,18], less attention has been devoted to studying the diversity in components and among protocells, which is basic property in all the life forms: cells generally involve a huge variety of components, while cells or replicators are diversified in all biological systems consisting of their population. In fact, one may naively expect that a simple replicating entity consisting of only a few molecular species would evolutionarily triumph as it can reproduce faster than a complex system using diverse components. Additionally, as replicators with the fastest division speed would dominate, coexistence of diverse replicators would not be expected. In addition to classical *invitro* experiments supporting this [19], several *insilico* artificial life models show dominance of such simple replicators [20,21].

This straight path of protocell population towards the fittest simple replicators seems to contradict with biodiversity in ecological populations [22-24], even at a cellular level [25,26]. Then, can both “*compositional*" diversity at an individual level and “*phenotypic*" diversity at the population level be present at “protocellular" stages, a primitive scenario of cellular evolution, in spite of the selective pressure for survival? Understanding how molecular mixtures give rise to reproducing cells and thereafter diversification both at individual and population levels will contribute to unravelling the intermediate phases or steps of molecules-to-population dynamics in the origin of life, and the dynamics of ecological populations from the underlying biochemical catalytic networks.

An important consequence of a system consisting of reproducing (proto)cells is competition for chemical resources needed for the growth. In most studies on the protocells or the origin of life, however, it is assumed that chemical resources for replication of biopolymers are supplied sufficiently. Indeed, in this case, a protocell consisting of fewer components, say hypercycles with three components, replicates faster, and quickly dominates the pool. As the catalytic activities of molecules and cell populations increase, however, nutrient depletion or resource competition is inevitable. The question we address in the present paper is how this resource limitation may lead to compositional diversity within a protocell and cell-to-cell diversification.

The effect of competition for a specific component is also demonstrated in protocells experimentally [27]. When only a single resource is provided, competition for the limited resource leads to survival of only the fittest protocell type. When *multiple* resources are competed for, then, do protocells diversify into distinct types specialized for the use of different resources, and is coexistence of diverse cell types possible? Here, we show through numerical simulation of a model of interacting protocells consisting of hypercycle reaction networks that such a transition to increased diversity occurs when available resources are limited. Further, we show that this diversification transition with the decrease of resource is understood as the transition from exponential to linear growth of cell population.

## Model

**Figure 1:**
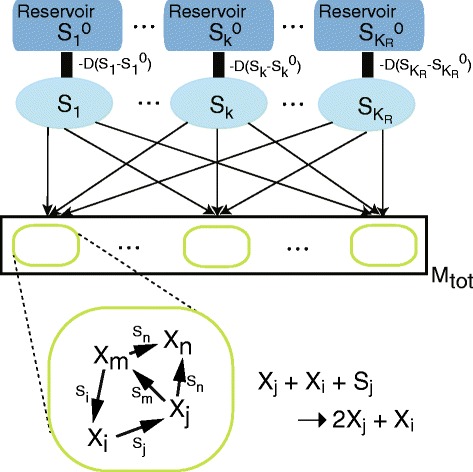
Schematic representation of our model. The system is composed of *M*_tot_ cells, each of which contains molecule species *X*_*j*_ (*j*=1,…,*K*_*M*_) that form a catalytic reaction network to replicate each *X*_*j*_. The cells share each resource *S*_*k*_ in a common medium, which is consumed by the replication of molecule *X*_*k*_. The resources flow into the common media for the cells from an external reservoir (environment) via diffusion $-D(S_{k}-{S_{k}^{0}})$ (*k*=1,…,*K*_*R*_), where ${S_{k}^{0}}$ is a randomly-fixed constant ${S_{k}^{0}} \in [0,M_{\text {tot}}]$ and *D* is the diffusion constant

For this purpose, we consider a simplified protocell model in which each molecule (*X*_*j*_;*j*=1,…,*K*_*M*_) replicates with the catalytic aid of *X*_*i*_, by consuming a corresponding resource (*S*_*j*_;*j*=1,…,*K*_*R*_). The outline of the model is as follows (Figure 1). There exist *M*_tot_ protocells, each consisting of *K*_*M*_ species of replicating molecules where some possibly have null population. Molecules of each species *X*_*j*_ are replicated with the aid of some other catalytic molecule *X*_*i*_, determined by a random catalytic reaction network(see below), by consuming a resource *S*_*j*_, one of the supplied resource chemicals *S*_*k*_(*k*=1,…,*K*_*R*_), as follows:

(1) $$ X_{j} + X_{i} + S_{j} \overset{c_{i}}\rightarrow 2X_{j} + X_{i}.  $$

For this reaction to replicate *X*_*j*_, one resource molecule is needed, and the replication reaction does not occur if *S*_*j*_<1. In this paper, we assume *K*_*M*_=*K*_*R*_, each resource species *S*_*j*_ corresponds to each molecule species *X*_*j*_. The case *K*_*M*_>*K*_*R*_ is discussed in the supplement, where a common resource molecule species is used for replication of multiple molecule species $X_{j_{1}},X_{j_{2}}..$. The reaction coefficient is given by the catalytic activity *c*_*i*_ of the molecule species *X*_*i*_, which is determined randomly as *c*_*i*_∈[0,1]. With each replication, error occurs with probability *μ* as shown below.

The resources (*S*_*j*_) diffuse sufficiently fast through a common medium for a population of protocells, so that replication of *X*_*j*_ can occur with corresponding reactions if *S*_*j*_ in the common medium is greater than one. From external reservoirs of concentrations ${S_{j}^{0}}$, the resources (*S*_*j*_) are supplied into the common medium by diffusion $-D(S_{j} - {S_{j}^{0}})$. *D* controls the degree of the resource competition, as the resource supply is limited with decreasing *D*.

The random catalytic reaction network is constructed as follows. For each molecule species, the density for the path of the catalytic reaction is given by *ρ*(which is fixed at 0.1) so that each species has *ρK*_*M*_ reactions on average. When a path exists between *X*_*i*_ and *X*_*j*_(*i*≠*j*), either is selected randomly as a catalyst for the replication of the other, while bidirectional connections are excluded so that *X*_*j*_ does not work as a catalyst for *X*_*i*_ if *X*_*i*_ is the catalyst for *X*_*j*_. Autocatalytic reactions in which *X*_*i*_ catalyzes the replication of itself are also excluded. Once chosen, the reaction network is fixed throughout each run of simulations, and the network is identical for all protocells of the population. Even though the underlying network is identical for all protocells, compositions in each protocell vary because of stochastic reactions, as well as structural changes explained below. By taking a different composition, each protocell can use a different part of the whole network for its growth.

Structural changes may occur during replication to alter monomer sequences of polymers and catalytic properties of the molecule. In the present model, this alteration is included as a random change to other molecular species during the replication process. When replication of *X*_*j*_ occurs, it is replaced by another molecule *X*_*l*_ (*l*≠*j*) with a probability *μ*. For simplicity, we assume that this error leads to all other molecule species with equal probability, *μ*/(*K*_*M*_−1) where *K*_*M*_ is the number of molecule species.

When the total number of molecules in each cell exceeds a given threshold *N*, the cell divides into two and partitions molecules in a totally random manner, irrespective of species, and one randomly chosen cell is removed from the system in order to fix the total number of cells at *M*_tot_.

Simulation is carried out as follows. We introduce discrete simulation steps. For each simulation step, we repeat the following procedures. For each cell, in a random order, we choose two molecules from the cell. If the pair of molecules, *X*_*i*_ and *X*_*j*_ is a catalyst and a replicator(*X*_*i*_ catalyzes the replication of *X*_*j*_), the reaction occurs with the given probability(*c*_*i*_), if *S*_*j*_≥1. When the reaction occurs, the new molecule of *X*_*j*_ is added into the cell and one molecule of the corresponding resource is subtracted to make *S*_*j*_→*S*_*j*_−1. Here, with a probability *μ*, a new molecule of *X*_*l*_(*l*≠*j*), instead of *X*_*j*_, is added into the cell as the structural change. If the total number of molecules in a cell exceeds a threshold *N*, the molecules are distributed into two daughter cells, while one cell, randomly chosen, is removed from the system. We also update each *S*_*a*_ to $S_{a}-D(S_{a}-{S^{0}_{a}}) (a=1,\ldots,K_{R})$.

## Results and discussion

### Diversification by decreasing resource supply in a catalytic reaction network

We simulated the model by changing the speed of resource supply *D*. The other parameters are fixed as *N*=1000, *K*_*M*_=*K*_*R*_=200 in this paper. For the moment, we also fix here *M*_tot_=100, and dependence on *M*_tot_ is discussed later. When the resources are supplied sufficiently fast (e.g., for *D*=1), a recursively growing state is established with a few molecular species, where the composition is robust against noise and perturbations by the division process.

**Figure 2:**
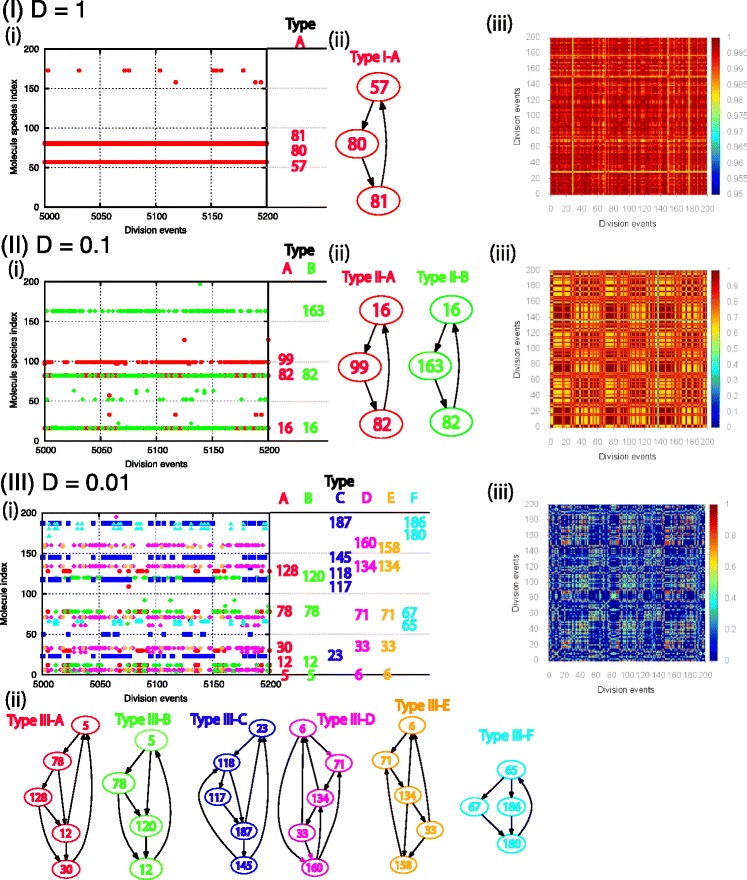
Compositions and types of reproducing protocells for (I) *D* = 1, (II) *D* = 0.1, and (III) *D* = 0.01. Compositions of molecule species are shown in successive 200 division events in the system with *K*_*M*_=*K*_*R*_. (i) At successive division events (abscissa), molecule species indices with more than 20 copy numbers in the dividing cell are marked. Cells are categorized into a few, or, several types, indicated by different colors, according to the majority set of molecule species. For each type, the indices of majority species are shown to the right of the figures. (ii)The catalytic network formed by the majority molecule species is shown for each cell type. Each numbered node corresponds to a molecule species, and the arrow from index *i* to *j* represents catalysis of *X*_*j*_ replication by *X*_*i*_. (iii) Similarities *H*_*ij*_ between cells *i* and *j*, as defined in the text, are plotted over 200 successive division events, using a color code from *H*_*ij*_∼1(red) to *H*_*ij*_∼0(dark blue). If the similarity is close to one, the composition is almost preserved by cell division, while zero similarity indicates that a reproduced cell has completely different composition. Intermediate values of similarity indicate the overlap in some molecule species between the cells, as in Type II-A and -B, Type III-A and -B, and Type III-D and -E. Parameters are *K*_*M*_=*K*_*R*_=200, *M*_tot_=100, *N*=1000, and *μ*=0.001

In this state, the (typically) three primary components form a three-component hypercycle (Figure 2(I)). There are a few hundred molecules out of *N*=1000 for each primary component, while most of the other molecules are null. Some species can appear by the random structural changes, but their number is typically less than a few. In some cases, molecular species being catalyzed by a member of the hypercycle increase their number(more than 20 copies), but the primary three components are robust enough. In other words, only a few molecule and reaction pathways among them are selected for their own growth from the overall structure of the catalytic network with *K*_*M*_ species. All the dividing cells adopt this three-component hypercycle, thus, there is neither compositional nor phenotypic diversity (Figure 2(I)).

To check cell reproduction fidelity, we introduced similarity between cells as follows: as each cellular state is characterized by the number of molecules of each chemical species $\vec {N}_{i} = (n_{1}, n_{2}, \ldots, n_{K_{M}})$, similarity is defined as the inner product of these composition vectors between two cell division events, i.e., $H_{\textit {ij}} = \vec {N}_{i} \cdot \vec {N}_{j}/(|\vec {N}_{i} || \vec {N}_{j} |)$ between the *i*-th and *j*-th division events. In the above case, the similarity between mother and daughter cells is close to one, implying high-fidelity reproduction.

As *D* decreases below 0.1 while keeping all the other parameters fixed, phenotypic diversity starts to increase. For example, two cell types(II-A,B) coexist in Figure 2(II) and consist of three-component hypercycles differing by one component. Both types divide with approximately equal speed and coexist over 10^2^ generations. In 200 successive division events(Figure 2(II) (iii)), one type has a similarity near unity(red), and the similarity of the other ranges between 0.6 and 0.7(yellow), implying that the two types mostly reproduce themselves with a small probability (approximately 0.01/division) of switching types. Over much longer generations, replication errors can produce different types capable of replacing the existing cell types.

As *D* decreases further, both the phenotypic and compositional diversity continue to increase. For *D*=0.01(Figure 2(III)), six cell types (*A*−*F*) appear. Each type forms a distinct hypercycle network in which the various species belonging to it catalyze the replication of each other. Here, some types (III −*A* and *B*, III −*D* and *E*) share some common molecular species, while the others do not. Similarities are approximately equal to unity(red) for some cells, while cell types that have the common chemical components(yellow to light blue) as well as cell types with completely orthogonal composition(dark blue) appear from time to time (Figure 2(III)(iii)). Also, the number of replicating chemical species in each cell is slightly increased(see Figure 2(III)(ii)). As *D* decreases to the order of 0.001, more cells with lower similarity appear, and compositional and phenotypic diversity further increase.

**Figure 3:**
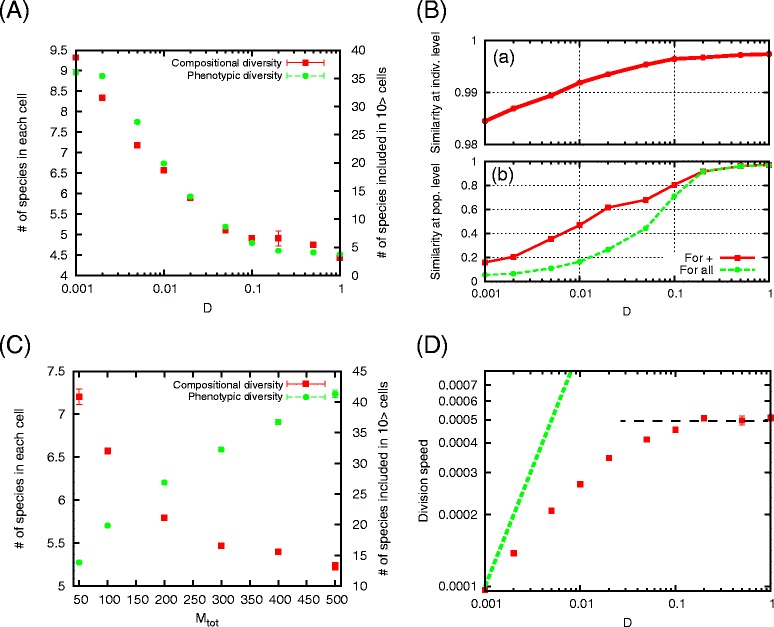
Quantitative dependence of diversity. **(A)** Compositional and phenotypic diversity plotted as a function of ***D***. For compositional and phenotypic diversity, the numbers of chemical species included in each cell (left ordinate), and in more than 10 cells out of *M*_tot_ cells (right ordinate) are shown. **(B)** (a) Average similarity of mother and daughter cells and (b) average similarity between cells in 10^4^ successive division events in 30 different networks, plotted against *D*. For (b), the average over cells with positive similarity(red) is shown in addition to the average for all cells (green). **(C)** Dependence of compositional (left ordinate) and phenotypic (right ordinate) diversity on the number of cells, *M*_tot_, with fixed *D*=0.01. **(D)** Average division speed as a function of *D*. For reference, a linear line with *D* is plotted. For *D*>0.2, an estimate of the division speed (∼0.0005) is also shown with a dotted line (see main text). Unless otherwise indicated, the data are obtained as the average over 10^5^ division events in 30 different networks. The parameters are *K*_*M*_=*K*_*R*_=200, *N*=1000, and *μ*=0.001

We statistically studied the quantitative dependence of diversity upon the parameter *D* using different network samples. As *D* decreases below ∼0.1, the compositional diversity of each protocell and the phenotypic diversity at the population level increase (Figure 3(A)). To confirm that the transition occurs independently of networks, we measure the number of species present in each cell as an index for compositional diversity, and that in cell population as an index for phenotypic diversity (for the latter we impose the condition that the molecule species exists for more than 10 cells, to discard species that happen to exist by the random structural changes), averaged in 30 different networks. Increase in the number of species in cell population more than that in each cell indicates the increase of phenotypic diversity in addition to that of compositional diversity. With the increase in both diversity, reproductive fidelity decreases both at an individual level (Figure 3B(a)) and at a population level, i.e., over all pairs of 10^4^ division events in 30 different networks (Figure 3B(b)). Protocells with different hypercycles start to appear below *D*∼0.1(see also Figure 2).

The transition to increased diversity generally occurs for sufficient resource diversity, i.e., for large *K*_*R*_, independent of reaction network choice, given *K*_*M*_=*K*_*R*_. The phenotypic diversity increases as ∼*K*_*R*_, but is bounded by the finite number of interacting cells, *M*_tot_ (Additional file 1). As *M*_tot_ increases, the number of coexisting cell-types increases, while the compositional diversity, i.e., the number of components in each cell type, decreases(see Figure 3(C)). This trade-off between compositional and phenotypic diversity suggests that each cell-type is specialized for fewer chemical components as the number of cell types increases.

Altogether, the data show transition behavior around *D*=*D*_*c*_∼0.1. Below *D*_*c*_, the division speed decreases, while above *D*_*c*_ it is approximately constant (Figure 3(D); see also below). This suggests that at the transition point the maximum inflow and consumption rates of resources are balanced. The maximum inflow rate is estimated as $D \bar {{S_{j}^{0}}}$, where $\bar {{S_{j}^{0}}}$ is a typical reservoir concentration. The intrinsic consumption rate by all cells is estimated to be $M_{\text {tot}} \bar {c_{i}} {p_{j}^{c}}$, where $\bar {c_{j}}$ is a typical catalytic activity, and ${p_{j}^{c}}$ is the probability of picking up a pair that replicates the molecule *X*_*j*_. When sufficient resources are available, the three-component hypercycle dominates, and ${p_{j}^{c}} \sim 1/9$. Therefore, the value of *D* at which the maximum inflow and consumption rates of resources are balanced is estimated as $D = M_{\text {tot}} \bar {c} /9\bar {{S_{j}^{0}}}$. Since *c*_*i*_ is distributed homogeneously in [0,1], its average value is 0.5; however the remaining components are typically biased to have higher catalytic activities, and $\bar {c}$ therefore typically ranges from 0.7 to 0.8. On the other hand, ${S_{j}^{0}}$ is distributed homogeneously in [0,*M*_tot_], so the simple average of $\bar {{S_{j}^{0}}}$ is 50 with *M*_tot_=100, but the remaining components are biased to have $\bar {{S_{j}^{0}}} \sim 70$. Thus the critical value of *D* is approximately 0.11−0.13. It is noteworthy that below *D*_*c*_ the division speed decreases more slowly than the inflow rate, as indicated by the line proportional to *D*(Figure 3(D)), suggesting that cells can utilize more diverse resources for growth by increasing the available number of resource species.

For *D*>0.2, sufficient resources are available, thus, the intrinsic reaction rate of the three-component hypercycle is the main determinant of the division speed. The probability, *p*_*c*_, for picking up a pair between which a catalytic reaction exists is ∼1/3. Using the above typical catalytic activity $\bar {c}$, the division speed is estimated as $2\bar {c}/3N$, which is approximately 0.0005 for *N*=1000 (See Figure 3(D)).

### Illustration by a simple case

Why does the transition to diversity occur with the decrease in resources? A simple case illustrates this diversity transition from dominance of a single type to coexistence of various types. Consider two types of cells that compete for resources. One type (*A*) consists of molecules *X* and *Y* while the other type (*B*) consists of molecules *X* and *Z*. The molecule species mutually catalyze the replication of each other to form a minimal hypercycle as follows:

(2) $$  X + Y + S_{X} \rightarrow 2X+Y, \,\,\, Y + X+ S_{Y} \rightarrow 2Y+ X  $$

and

(3) $$  X + Z + S_{X} \rightarrow 2X+Z, \,\,\, Z + X + S_{Z} \rightarrow 2Z+ X.  $$

We denote the intrinsic catalytic activities of *X*, *Y*, and *Z*, respectively, by *c*_*X*_, *c*_*Y*_ and *c*_*Z*_. Each reaction to synthesize *X*, *Y*, and *Z* utilizes the resource *S*_*X*_, *S*_*Y*_, and *S*_*Z*_, respectively, which are shared by cells.

While rate equations of the simple case are investigated in details both analytically and numerically in Additional file 1, here we show results of stochastic simulations of the case in which the setup is given in the same way as the original model and present intuitive explanations of the result. We consider *M*_tot_ cells, each of which consists of either pair of *X*,*Y* or *X*,*Z*, corresponding to type *A* or *B*, respectively. The resources *S*_*X*_, *S*_*Y*_, and *S*_*Z*_ flow from the outer reservoir with diffusion constant *D*. For simplicity, we assume *c*_*X*_=*c*_*Y*_=*c*_*Z*_=1, and ${S^{0}_{X}} = {S^{0}_{Y}} = {S^{0}_{Z}} = M_{\text {tot}}$. The diffusion constants are identical for *X*, *Y* and *Z*.

**Figure 4:**
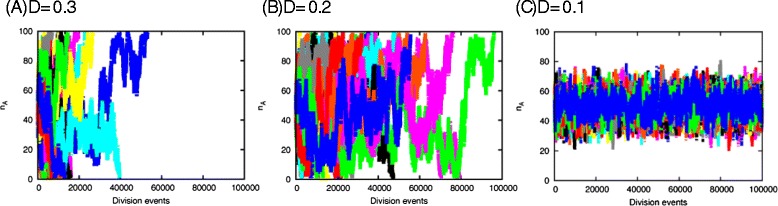
Time evolution of *n*_*A*_ for (A) D = 0.3 (B) D = 0.2 and (C) D = 0.1. The initial state is given as *n*_*A*_=*n*_*B*_=*M*_tot_/2, and in each type *A* and *B*, we randomly distribute molecules, *X* and *Y*, and *X* and *Z*, respectively, with equal probabilities. Here, *M*_tot_ = 100, *N*=1000. Different colors indicate different simulation runs

The evolution of the number of type *A*, *n*_*A*_, for different values of *D* is given in Figure 4. For *D*=0.3, either *A* or *B* is extinct after a relatively short time span. Around *D*=0.2, the transient time before extinction increases, and the two types coexist over more than 10^5^ division events for *D*=0.1.

**Figure 5:**
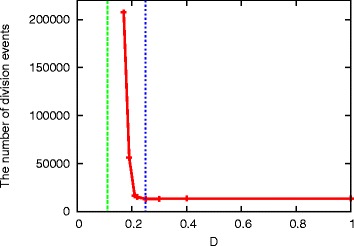
Average number of division events, as a function of *D*, to achieve a state where either *A* or *B* cells dominates the system. Here, *M*_tot_ = 100, *N*=1000. The dotted lines show *D* ^∗^=1/4 and *D* ^+^=1/9

The average number of division events to achieve the dominant state as a function of *D* is given in Figure 5. It is clear that the coexistence time starts to increase around *D*^∗^=0.25, which is consistent with the point where the resources start to compete. In the stationary state without competition, the number of *X* in each cell is equal to the number of *Y* in type *A*, and the number of *Z* in type *B*, i.e., ${N_{X}^{A}} = {N_{Y}^{A}}$ and ${N_{X}^{B}} = {N_{Z}^{B}}$ where ${N_{i}^{I}}$ is the number of *i*(*i*=*X*,*Y*,*Z*) in type *I*(*I*=*A*,*B*). Thus, the point where *S*_*X*_ is limited is given by $D^{*} {S_{X}^{0}} = \frac {1}{4} M_{\text {tot}}$, therefore, *D*^∗^=1/4 for ${S_{X}^{0}} = M_{\text {tot}}$.

As *D* is further decreased, the coexistence of types *A* and *B* is achieved when *S*_*Y*_ and *S*_*Z*_ are also limited and competed for by cells. In this case, the coexistence is stable as analyzed in the Additional file 1. In the smaller limit of D where all the resources are limited, the steady state approaches ${N_{X}^{A}} = 2 {N_{Y}^{A}}$, ${N_{X}^{B}} = 2{N_{Z}^{B}}$. This gives *D*^+^*S*^0^=*M*_tot_/9. For *S*^0^=*M*_tot_, *D*^+^∼0.11. This is also consistent with the observation in Figure 4.

The transition from dominance to coexistence states can be explained as follows. When all the resources are supplied sufficiently, the population of each cell type grows exponentially, where the growth rate is proportional to the number *n*_*A*(*B*)_ of each cell type *A* and *B*, i.e., *dn*_*A*(*B*)_/*dt*∝*γ*_*A*(*B*)_*n*_*A*(*B*)_, and the proportionality coefficient, *γ*_*A*(*B*)_, depends on resource abundances *S*_*Y*(*Z*)_. In this case, Darwinian selection works so that the stable solution to the equations is a dominance of the fittest type with a larger *γ*. This selection process works as long as the resources *S*_*Y*(*Z*)_ are sufficient. The competition of the single common resource *S*_*X*_ results in a dominance of the single type. However, when all the resources are limited, competition for the available resources *S*_*Y*(*Z*)_ among *n*_*A*(*B*)_ cells decreases *γ*_*A*(*B*)_ so that it is inversely proportional to *n*_*A*(*B*)_, respectively. The population dynamics are, therefore, represented by *dn*_*A*(*B*)_/*dt*∝*c*_*A*(*B*)_, with constant *c*_*A*(*B*)_, when the maximum inflow is decreased to balance with the rate of resource consumption. In this form, the solution with two coexisting types is known to be stable [9,28] as shown in Additional file 1. The transition to diversity in composition and phenotypes in the original model is based on this change from exponential to linear growth due to resource limitation. On the other hand, the above argument on the diversity transition supports the generality of our result, as the number growth in chemical components by catalytic reactions changes from exponential to linear with the decrease in resources.

## Conclusions

In summary, we show that coexistence of diverse compositions and cell-types is the result of competitions for a variety of limited resources. We find that a transition to diversity occurs both in chemical compositions and in protocell types, as the resource supply is decreased, when the maximum inflow and consumption of resources are balanced. By a simple case, we also demonstrate that the diversity is based on the change from exponential to linear growth due to resource limitation.

When life originated, a set of diverse, self-replicating catalytic polymers(replicators) would emerge from the primordial chemical mixture, to make a reproducing protocell. Although the importance of molecular and protocellular diversity has been noted by Dyson [5], their origins are not well addressed, especially as compared with diversity in ecological systems. Our results indicate that competition for a variety of limiting resources can be a strong driving force to diversify intracellular dynamics of a catalytic reaction network and to develop diverse protocell types in a primitive stage of life. Indeed, it is natural that diverse chemical resources are available in the environment, and competition for resources increases as the protocells reproduce and more cells compete. Thus, diversification in composition and protocell types is an inevitable outcome.

According to our results, the diversification is understood as a kind of phase transition in population dynamics with decreased resource. In ecology, the niche dimension hypothesis in plant communities was proposed, in which the number of coexisting species increases with the number of limiting factors, while diversity decreases with greater resource abundance [29-31]. Our results suggest that such population dynamics in ecology are also possible with primitive mixtures of catalytic molecules competing for a variety of resources, even without the need for genetic changes as in speciation.

## Additional file

Additional file 1 **Supplementary material for transition to diversification by competition for multiple resources in a catalytic reaction network.**
