# Supplementary material for: Transition to diversification by competition for multiple resources in catalytic reaction networks
Source: J Syst Chem. 2015 Apr 2;6(1):5. doi: 10.1186/s13322-015-0010-1 (PMC4412551; doi:10.1186/s13322-015-0010-1)
Supplement: Supplementary file 1 — Supplementary material for transition to diversification by competition for multiple resources in a catalytic reaction network. [file 13322_2015_10_MOESM1_ESM.pdf]

Supplementary Material for Transition to diversification by competition for multiple resources in a catalytic reaction network

Atsushi Kamimura[1] and Kunihiko Kaneko[1]

[1]Department of Basic Science, The University of Tokyo, 3-8-1, Komaba, Meguro-ku, Tokyo 153-8902, Japan

(Dated: March 9, 2015)

I. A SIMPLE CASE - MUTUALLY CATALYTIC MOLECULES -

A. Reactions

Let us consider two types of cells. One type, denoted by A, consists of molecule species X and Y. The other type, B, consists of molecule species X and Z. The molecule species mutually catalyze the replication of each other to form a minimal hypercycle as follows:

$$X + Y + S_X \to 2X + Y, \quad Y + X + S_Y \to 2Y + X$$

and

$$X + Z + S_X \to 2X + Z, \quad Z + X + S_Z \to 2Z + X.$$

We denote the intrinsic catalytic activities of X, Y, and Z, respectively, by c_X, c_Y and c_Z. Each reaction to synthesize X, Y, and Z utilizes the resource S_X, S_Y, and S_Z, respectively, which are shared by cells.

The intrinsic reaction rates for replicating the molecule species X are given by

$$F_X^A = V_A f_X^A s_X, \quad F_X^B = V_B f_X^B s_X,$$

respectively, for cell type A and B. Here, V_A, and V_B are the volumes of cell types A and B, respectively, and $f_X^A = c_Y x_X^A x_Y^A, f_X^B = c_Z x_X^B x_Z^B$, where $x_i^I = N_i^I / V_I (i = X, Y, Z; I = A, B)$ and N_i^I is the number of molecule species i. The rates of replicating Y and Z are, respectively, given by

$$F_Y^A = V_A f_Y^A s_Y, \quad F_Z^B = V_B f_Z^B s_Z$$

Here, $f_Y^A = c_X x_X^A x_Y^A, f_Z^B = c_X x_X^B x_Z^B$. $s_i = S_i / S_i^0 (i = X, Y, Z)$ is the normalized concentration of the resource where S_i is the concentration and S_i^0 is introduced to normalize S_i to one when $S_i = S_i^0$.

The rate equations of cell-types A and B for molecule species X are written as,

$$\frac{dN_X^A}{dt} = F_X^A = V_A f_X^A s_X, \frac{dN_X^B}{dt} = F_X^B = V_B f_X^B s_X, \tag{1}$$

and, for Y and Z,

$$\frac{dN_Y^A}{dt} = F_Y^A = V_A f_Y^A s_Y, \frac{dN_Z^B}{dt} = F_Z^B = V_B f_Z^B s_Z. \tag{2}$$

The dynamics of resources S_X, S_Y, and S_Z are respectively written as

$$\frac{dS_X}{dt} = -\left(V_A f_X^A + V_B f_X^B\right) s_X + DS_X^0(1 - s_X), \tag{3}$$

$$\frac{dS_Y}{dt} = -V_A f_Y^A s_Y + DS_Y^0(1 - s_Y), \tag{4}$$

$$\frac{dS_Z}{dt} = -V_B f_Z^B s_Z + DS_Z^0(1 - s_Z). \tag{5}$$

In the steady state, the value of s_X, $\bar{s}_X$, is written as,

$$\bar{s}_X = \frac{DS_X^0}{\left(V_A f_X^A + V_B f_X^B\right) + DS_X^0}. \tag{6}$$

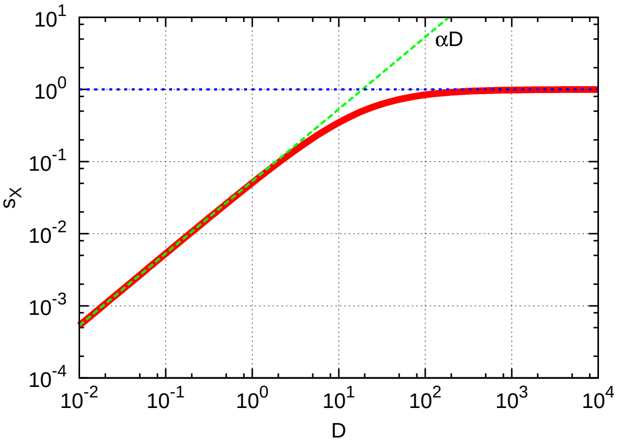

FIG. 1: The value of $\bar{s}_X$ as a function of D. The parameters are $c_Y = c_Z = 1$, and $S_X^0 = 1000$. The x_i^I is fixed to $1/2 (i = X, Y, Z, I = A, B)$, and $V_A = V_B = 750 M_{\rm tot}/2$ with $M_{\rm tot} = 100$. The line αD is also shown with $\alpha = S_X^0 / \left(V_A f_X^A + V_B f_X^B \right)$.

In Fig. 1, we show $\bar{s}_X$ as a function of D with a set of parameters. For large D, $\bar{s}_X \to 1$. As D is decreased, $\bar{s}_X$ starts to decrease and deviates from one. For smaller D, $\bar{s}_X$ decreases linearly as αD.

Similarly, for resources S_Y and S_Z the steady state values are written as

$$\bar{s}_Y = \frac{D S_Y^0}{V_A f_Y^A + D S_Y^0}, \qquad \bar{s}_Z = \frac{D S_Z^0}{V_B f_Z^B + D S_Z^0}. \tag{7}$$

The dynamics of each molecule species changes with the diffusion constant D. When D is sufficiently large, $\bar{s}_X$ is approximately one. As D decreases and the resource is limited, $\bar{s}_X$ deviates from one, and the maximum inflow rate of the resource is given by $J_X = D S_X^0 (1 - \bar{s}_X)$. In each range of D, we refer to $(\mathrm{X - i})$:S_X is sufficiently supplied, and $(\mathrm{X - ii})$: S_X is limited.

For each range, the right-hand-sides of the rate equations (1) can be written as

$$(F_X^A, F_X^B) = \begin{cases} (V_A f_X^A, V_B f_X^B) & \text{for } (\mathrm{X - i}) \\ (r_A J_X, r_B J_X) & \text{for } (\mathrm{X - ii}) \end{cases}$$

where r_A and $r_B = 1 - r_A$ are the ratios of the resource being distributed to cell types A and B.

For the resources S_Y and S_Z, we assume the range changes simultaneously for both of Y and Z because, otherwise, either type with a limited resource will vanish; we refer to $(\mathrm{YZ - i})$: S_Y and S_Z are sufficiently supplied, and $(\mathrm{YZ - ii})$: S_Y and S_Z are limited. For each range, the right-hand-sides of the rate equations (2) can be written as

$$F_Y^A = \begin{cases} V_A f_Y^A & (\mathrm{YZ - i}) \\ J_Y & (\mathrm{YZ - ii}) \end{cases}, F_Z^B = \begin{cases} V_B f_Z^B & (\mathrm{YZ - i}) \\ J_Z & (\mathrm{YZ - ii}) \end{cases}$$

where $J_Y = D S_Y^0 (1 - \bar{s}_Y)$ and $J_Z = D S_Z^0 (1 - \bar{s}_Z)$. As will be shown in subsequent subsections, we investigate four cases for the conditions $(X, YZ) = (\mathrm{i}, \mathrm{i}), (\mathrm{ii}, \mathrm{i}), (\mathrm{i}, \mathrm{ii})$ and $(\mathrm{ii}, \mathrm{ii})$.

The volume V_A, V_B of each cell increases in proportional to the numbers of molecules as

$$V_A = \frac{1}{R}(N_X^A + N_Y^A), V_B = \frac{1}{R}(N_X^B + N_Z^B),$$

where R is a constant.

Then the increase in volumes is given by

$$\frac{dV_A}{dt} = \frac{1}{R}(F_X^A + F_Y^A), \frac{dV_B}{dt} = \frac{1}{R}(F_X^B + F_Z^B),$$

Let us suppose that a cell divides when its volume exceeds a certain critical value and that the total volume of all cells is restricted by some constant $\sum V_k = V_T = const$. Then the volume fraction of each type A and B follows

$$\frac{dv_A}{dt} = \frac{1}{RV_T}(F_X^A + F_Y^A) - \frac{v_A}{R}\sum \frac{F^I}{V_T},$$
$$\frac{dv_B}{dt} = \frac{1}{RV_T}(F_X^B + F_Z^B) - \frac{v_B}{R}\sum \frac{F^I}{V_T}.$$

where $v_I = V_I/V_T (I = A, B)$, $F_I = \sum F_i^I$.

B. The case in which all the resources are sufficiently available: condition (X, YZ) = (i, i)

For the condition $(X, YZ) = (\mathrm{i}, \mathrm{i})$, when all resources are sufficiently available, the increases in molecule species are written as

$$F_X^A = V_A f_X^A, F_Y^A = V_A f_Y^A, F_X^B = V_B f_X^B, F_Z^B = V_B f_X^B,$$

where

$$f_X^A = c_Y x_X^A x_Y^A, f_X^B = c_Z x_X^B x_Z^B, f_Y^A = c_X x_X^A x_Y^A, f_Z^B = c_X x_X^B x_Z^B,$$

and $x_i^I = N_i^I/V_I (I = A, B, i = X, Y, Z)$. Thus,

$$\frac{dv_A}{dt} = \frac{f^A}{R} v_A - \frac{v_A}{R}\sum_I v_I f^I,$$
$$\frac{dv_B}{dt} = \frac{f^B}{R} v_B - \frac{v_B}{R}\sum_I v_I f^I,$$

where $f^A = f_X^A + f_Y^A, f^B = f_X^B + f_Z^B$. The stationary states give

$$v_A f^A = v_A \left(v_A f^A + v_B f^B\right),$$
$$v_B f^B = v_B \left(v_A f^A + v_B f^B\right).$$

The solutions to these equations are $(v_A, v_B) = (1, 0)$ or $(0, 1)$.

By writing $v_A = \delta$ and $v_B = 1 - \delta$, linearization around $(v_A, v_B) = (0, 1)$ gives

$$\frac{d\delta}{dt} = \frac{f_A}{R}\delta - \frac{\delta}{R}(\delta f^A + (1-\delta) f^B)$$
$$= \frac{\delta}{R}(f_A - f_B).$$

Thus, the fixed point $(v_A, v_B) = (0, 1)$ is stable when $f_B > f_A$. On the other hand, $(v_A, v_B) = (1, 0)$ is stable when $f_A > f_B$. Hence, the fittest cell type, i.e., that with larger f_i(i=A or B) dominates the population.

C. The case S_X is limited: the condition (X, YZ) = (ii, i)

For the condition $(X, YZ) = (\mathrm{ii}, \mathrm{i})$, when S_X is limited,

$$F_X^A = r_A J_X, F_X^B = r_B J_X, F_Y^A = V_A f_Y^A, F_Z^B = V_B f_Z^B,$$

where J_X is the maximum inflow of S_X and r_A and $r_B = (1 - r_A)$ are the ratios of S_X distributed into A and B. Then,

$$\frac{dv_A}{dt} = \frac{1}{R}\left(r_A j_X + f_Y^A v_A\right) - \frac{v_A}{R}\left(j_X + f_Y^A v_A + f_Z^B v_B\right),$$
$$\frac{dv_B}{dt} = \frac{1}{R}\left(r_B j_X + f_Z^B v_B\right) - \frac{v_B}{R}\left(j_X + f_Y^A v_A + f_Z^B v_B\right),$$

where $j_X = J_X/V_T$. The stationary condition is written as

$$v_A \left\{ j_X + f_Y^A (v_A - 1) + f_Z^B v_B \right\} = r_A j_X,$$
$$v_B \left\{ j_X + f_Z^B (v_B - 1) + f_Y^A v_A \right\} = r_B j_X.$$

The stationary condition has solutions $(v_A, v_B) = (1,0), (0,1)$. The solution $(v_A, v_B) = (1,0)$ satisfies the condition with $r_A = 1$. Linearizing the rate equation around the fixed point $(1,0)$ by writing $v_A = 1 - \delta$, we get

$$\frac{d\delta}{dt} = -\frac{1}{R}\left(f_Y^A - f_Z^B + j_X\right)\delta.$$

When $f_Y^A \geq f_Z^B$, the solution is stable. The solution $(v_A, v_B) = (0,1)$ satisfies the equation when $r_A = 0$. By linearizing the rate equation around the fixed point $(0,1)$ and writing $v_A = \delta$, we obtain

$$\frac{d\delta}{dt} = -\frac{1}{R}\left(f_Z^A - f_Y^A + j_X\right)\delta.$$

When $f_Z^B \geq f_Y^A$, the solution is stable.

For the case $f_Y^A = f_Z^B$, the equation is written as

$$\frac{d\delta}{dt} = -\frac{j_X}{R}\delta,$$

thus, both solutions are stable.

D. The case S_Y and S_Z are limited: condition $(X, YZ) = (\mathrm{i}, \mathrm{ii})$

For the condition $(X, YZ) = (\mathrm{i}, \mathrm{ii})$ when S_Y and S_Z are limited, increases of molecule species are written as

$$F_X^A = V_A f_X^A, F_X^B = V_B f_X^B, F_Y^A = J_Y, F_Z^B = J_Z.$$

Then,

$$\frac{dv_A}{dt} = \frac{1}{R}\left(f_X^A v_A + j_Y\right) - \frac{v_A}{R}\left(f_X^A v_A + f_X^B v_B + j_Y + j_Z\right),$$
$$\frac{dv_B}{dt} = \frac{1}{R}\left(f_X^B v_B + j_Z\right) - \frac{v_B}{R}\left(f_X^A v_A + f_X^B v_B + j_Y + j_Z\right).$$

The stationary state gives

$$(f_X^A - f_X^B)v_A^2 + (-f_X^A + f_X^B + j_Y + j_Z)v_A - j_Y = 0.$$

When $f_X^A = f_X^B$, this equation reduces to a solution

$$v_A = v_A^0 = \frac{j_Y}{j_Y + j_Z}, v_B = v_B^0 = \frac{j_Z}{j_Y + j_Z}.$$

By linearizing the dynamics around the fixed point as $v_A = v_A^0 + \delta$, we get

$$\frac{d\delta}{dt} = -\frac{1}{R}\left(j_Y + j_Z\right)\delta,$$

and the solution is stable.

When $f_X^A > f_X^B$, the equation has a fixed-point solution with coexistence of A and B as

$$v_A = v_A^0 = \frac{1}{2\left(f_X^A - f_X^B\right)}\left\{f_X^A - f_X^B - j_Y - j_Z + \sqrt{(f_X^A - f_X^B - j_Y - j_Z)^2 + 4j_Y(f_X^A - f_X^B)}\right\}.$$

As will be shown below, $0 < v_A^0 < 1$, so that the two types coexist: First, since $f_X^A - f_X^B > 0$, $v_A^0 > 0$. Then the difference in the numerator and denominator in the expression of v_A^0 is given by

$$(numerator) - (denominator) = -(f_X^A - f_X^B + j_Y + j_Z) + \sqrt{(f_X^A - f_X^B - j_Y - j_Z)^2 + 4j_Y(f_X^A - f_X^B)}.$$

Here,

$$|f_X^A - f_X^B + j_Y + j_Z|^2 - \left\{(f_X^A - f_X^B - j_Y - j_Z)^2 + 4j_Y(f_X^A - f_X^B)\right\} = 4j_Z(f_X^A - f_X^B) > 0,$$

so that the denominator is greater than the numerator. Thus, $v_A^0 < 1$. Therefore, $0 < v_A^0 < 1$.

By writing $v_A = v_A^0 + \delta$, and linearizing the dynamics around the fixed point, we obtain

$$\frac{d\delta}{dt} = -\left\{(f_X^A - f_X^B)(2v_A^0 - 1) + j_Y + j_Z\right\}\delta.$$

The solution is stable if $(f_X^A - f_X^B)(2v_A^0 - 1) + j_Y + j_Z > 0$. For example, if $f_X^A > f_X^B$ and $j_Y \geq j_Z$,the condition is satisfied.

When $f_X^B > f_X^A$, the stationary condition is

$$(f_X^B - f_X^A)v_B^2 + (-f_X^B + f_X^A + j_Y + j_Z)v_B - j_Z = 0,$$

for v_B. The fixed-point solution is obtained in the same way as in the case $f_X^A > f_X^B$, by replacing A with B, and Y with Z.

E. All the S_X, S_Y and S_Z are limited: condition $(X, YZ) = (\mathrm{ii}, \mathrm{ii})$

For the condition $(X, YZ) = (\mathrm{ii}, \mathrm{ii})$, when all resources are limited,

$$F_X^A = r_A J_X, F_X^B = r_B J_X, F_Y^A = J_Y, F_Z^B = J_Z.$$

Then,

$$\frac{dv_A}{dt} = \frac{1}{R}(r_A j_X + j_Y) - \frac{v_A}{R}(j_X + j_Y + j_Z),$$
$$\frac{dv_B}{dt} = \frac{1}{R}(r_B j_X + j_Z) - \frac{v_B}{R}(j_X + j_Y + j_Z).$$

The steady state gives

$$v_A^0 = \frac{r_A j_X + j_Y}{j_X + j_Y + j_Z}, v_B^0 = \frac{r_B j_X + j_Z}{j_X + j_Y + j_Z}.$$

Again by writing $v_A = v_A^0 + \delta$,

$$\frac{d\delta}{dt} = -\frac{j_X + j_Y + j_Z}{R}\delta,$$

follows, and the coexisting state is stable.

F. Numerical simulations

In this subsection, we present results of numerical simulations of the rate equations for the continuous concentration variables to show whether competition of limited resources results in dominance or coexistence of cells of type A and B. The results for stochastic simulations are shown in the main text.

We consider M_{tot} cells of type A or B. The dynamics of the number N_i^I of molecule species $i(i = X, Y, Z)$ in a cell-type $I(I = A, B)$ is written as eqs. (1) and (2). The dynamics of each resource $S_i(i = X, Y, Z)$ is given by eqs. (3)-(5).

As an initial distribution of cell types, types A and B are each represented at 50%. In each type, the numbers N_X, N_Y in type A, and N_X, N_Z in type B are randomly assigned. A cell divides when $V_K = \sum_i N_i^K$ exceeds a threshold $V_{\max}$. At the division, the number of each molecule species N_i^K is divided between the two daughter cells with a normal distribution with average $N_i^K/2$ and variances $N_i^K/4$. The normal distribution approximates the random partition of molecules into two daughter cells.

The average time required for the initial condition to reach a state in which either of the types is extinct is shown in Fig. 2. The parameters approximately correspond to those of Fig. 1. The time drastically increases around $D^* = 100$,

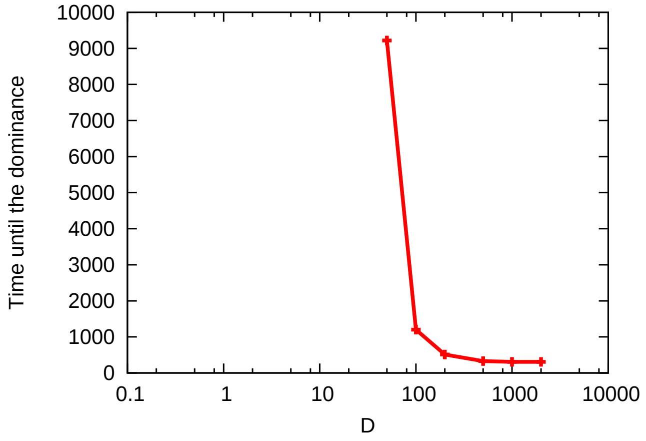

FIG. 2: Average time to a state in which either A or B dominates. Here, $M_{\rm tot} = 100$, $V_{\rm max} = 1000$, $S_X^0 = S_Y^0 = S_Z^0 = 1000$.

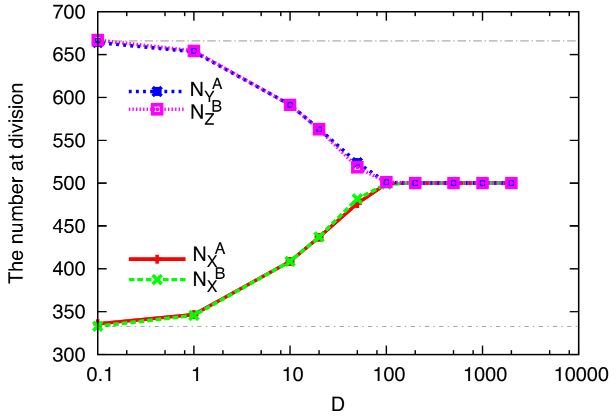

FIG. 3: Average number of N_X, N_Y, and N_Z at division events. The initial state is given as $n_A = n_B = M_{\rm tot}/2$, and in each type A and B, we randomly distribute molecules X and Y, and X and Z, respectively, with equal probabilities.

which is consistent with the point where the resource S_X starts to be limited in Fig. 1. Correspondingly, the average numbers of N_X, N_Y and N_Z at division events start to deviate below that point(Fig. 3), and available resources show deviations between S_X and S_Y or S_Z below that point(Fig.4).

At the point $D = D^*$, consumption and inflow of resources are balanced and S_X starts to compete. The condition for balance in S_X is

$$\frac{M_{\rm tot} V}{4} \frac{S_X}{S_X^0} = D^* \left(S_X^0 - S_X \right),$$

where V denotes the average volume of cells. By substituting $V = 750$, $S_X = 850$, $S_X^0 = 1000$, $M_{\rm tot} = 100$, D^* is estimated as ~ 100.

For S_Y and S_Z, competition starts roughly when the inflow is half that of S_X because approximately half of $M_{\rm tot}$ cells consume the respective resource. This suggests that below $D = 0.5D^*$, all the resources are limited; thus, two types of cells coexist. This is consistent with our numerical observations that below $D = 40$, the types coexist.

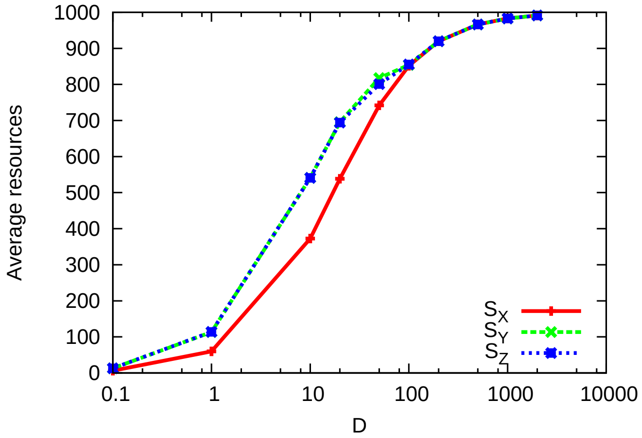

FIG. 4: Average resources S_X, S_Y and S_Z. Here, $M_{\rm tot} = 100$, $V_{\rm max} = 1000$, $S_X^0 = S_Y^0 = S_Z^0 = 1000$.

II. WHEN DIFFERENT MOLECULE SPECIES CONSUME COMMON RESOURCES : THE CASE WITH $K_R < K_M$

In this section, we investigate the case with $K_R < K_M$ where plural resource species are commonly used for the replication of different molecules X_i.

A. Model

In the reaction

$$X_j + X_i + S_{\hat{j}} \to 2X_j + X_i,$$

the correspondence between molecule X_j and resource $S_{\hat{j}}$ is randomly assigned and fixed throughout simulations. Hence, each resource species is used commonly for K_M/K_R reactions on the average.

B. Results

We show the number of molecule species in each cells (compositional diversity) and that in 10 or more cells (phenotypic diversity), respectively, in Fig. 5(A) for $K_R = 1$, i.e., a single resource is consumed to replicate all the molecule species X_i $(i = 1, ..., K_M)$. In this case, neither compositional nor phenotypic diversity increases as the diffusion constant D decreases.

When two resource species are available($K_R = 2$; Fig. 5(B)), the phenotypic diversity increases as D decreases, but a clear increase is not discernible in the compositional diversity. In the case of small K_R, the randomly determined reservoir concentrations, $S_0^i \in [0, M_{\rm tot}](i = 1, 2)$, are also relevant parameters to determine the point at which the resources become limited, in addition to the diffusion constant. Here, we also show the results for fixed $S_i^0 = M_{\rm tot}(i = 1, 2)$. In contrast to the $K_M = K_R$ case, there is an increase in phenotypic diversity for $D < 0.1$, while even below the point, the number of remaining chemical species is approximately constant and does not increase as D is decreased. This result indicates that the diversity is bounded by the number of resource species: coexistence of at most two cell types is possible.

For larger K_R ($K_R = 10$ in Fig. 5(C), and $K_R = 100$ in Fig. 5(D)), both compositional and phenotypic diversity increase as D is decreased because K_R is sufficiently large so that it does not effectively restrict the number of cell types. The phenotypic diversity, i.e., the number of coexisting cell types increases as $\sim K_R$, but the increase is saturated for larger K_R (see Fig. 5), which is also bounded by $M_{\rm tot}$. Indeed, as $M_{\rm tot}$ is increased, the number increases (see below).

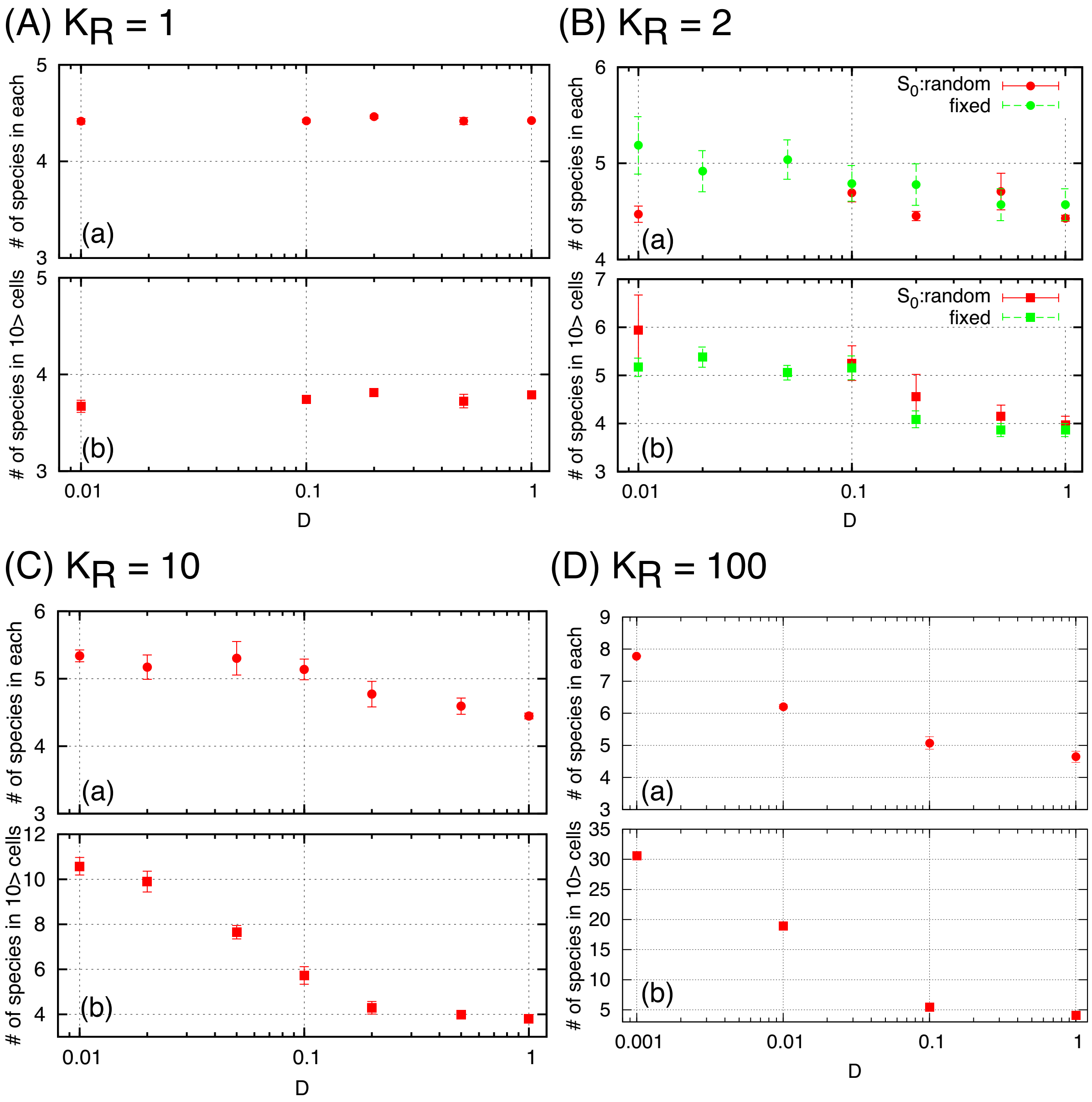

FIG. 5: Compositional and phenotypic diversity plotted as a function of D for (A) $K_R = 1$, (B) $K_R = 2$, (C) $K_R = 10$, and (D) $K_R = 100$. Compositional and phenotypic diversity are computed in the same way as in the main text, i.e., as the numbers of chemical species included in (a) each cell, and in (b) more than 10 cells out of $M_{\rm tot}$ cells are shown, respectively. For (B)$K_R = 2$, the data for two cases are shown: $S_0^i(i = 1, 2)$ is determined randomly between 0 and $M_{\rm tot}$, and both $S_0^i(i = 1, 2)$ are fixed to $M_{\rm tot}$ All the data are obtained as the average over 10^5 division events in 5 different networks with $M_{\rm tot} = 100$, $K_M = 200$, $N = 1000$, and $\mu = 0.001$.

III. DEPENDENCE ON $M_{\rm tot}$

We investigated the dependence of the diversity on the number of interacting cells, $M_{\rm tot}$, in the case $K_M = K_R$. The number of molecule species in each cells (compositional diversity) and that in 10 or more cells (phenotypic diversity) for $M_{tot} = 100, 200$, and 300 is shown in Fig. 6.

While both measures of diversity increase for each $M_{\rm tot}$, as D is decreased, the increment depends on $M_{\rm tot}$. The increment in compositional diversity decreases as $M_{\rm tot}$ is increased. On the other hand, the increment in phenotypic diversity with the decrease in D increases as $M_{\rm tot}$ is increased. As shown in Fig. 3(C) of the main text, the two types

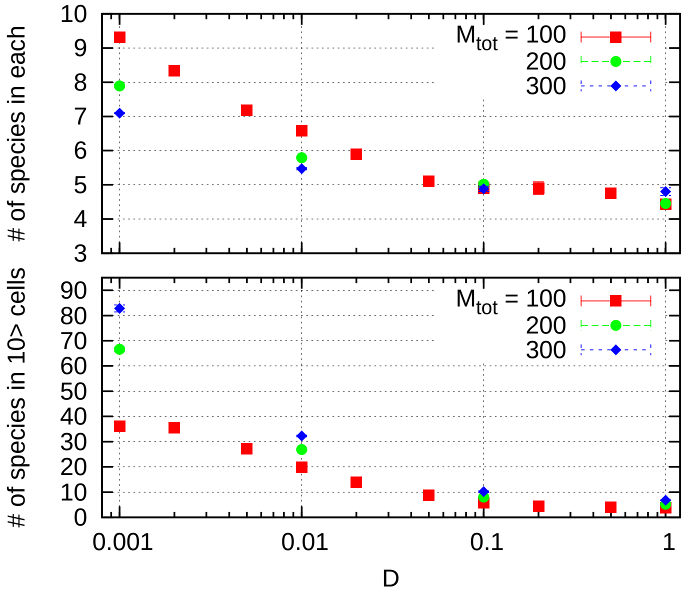

FIG. 6: Compositional and phenotypic diversity for $M_{\rm tot} = 100, 200$, and 300. The data are obtained as the average over 10^5 division events in 30 different networks with $K_M = K_R = 200$, $N = 1000$, and $\mu = 0.001$

of diversity clearly show opposite dependence on $M_{\rm tot}$ for fixed D.

As discussed in the previous section, multiple cell types (up to K_R) can coexist; thus, more types of cells can be present and are not eliminated from the system as $M_{\rm tot}$ is increased, which results in the increase in phenotypic diversity. On the other hand, as the number of cell types increases, a greater number of resource species are competitive because the cell population can consume more resource species. This leads each individual cell to be more specialized with fewer components, which results in suppression of the increase in compositional diversity.

IV. SUPPLEMENTARY FIGURES

Figs 7 and 8 show similarities H_{ij} among a period of division events, where the cell indices given by the $x-$ and $y-$ axes are rearranged so that the same (similar) types are clustered. The data are identical to the similarities represented in Fig. 2(II)(iii) and (III)(iii) of the main text, but the plots are given after rearrangement of cell indices.

Types II-A and II-B are clustered as shown at the top of Fig. 7. The similarities between cells of the same type have values close to 1, while types A and B have similarities around 0.6.

The types from III-A to III-F are clustered at the top of Fig. 8. The similarities between cells of the same type have values close to 1, and those between types A and B, and between D and E have positive values. For other pairs, the values are almost orthogonal.

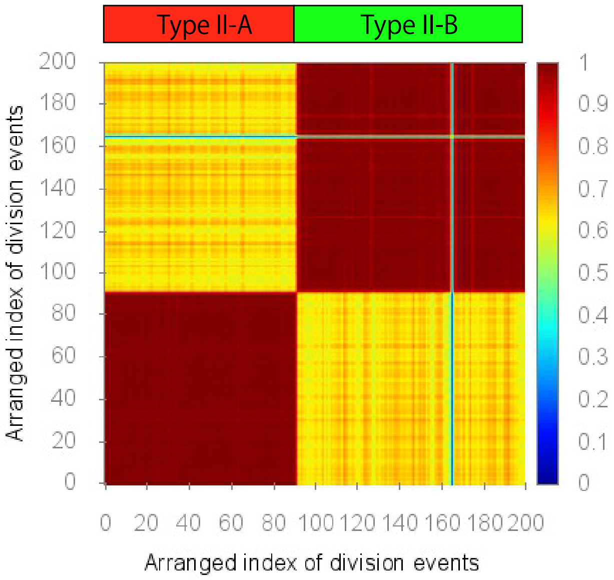

FIG. 7: The indices are arranged from Fig. 2(II)(iii) in the main text to categorize into each type A and B.

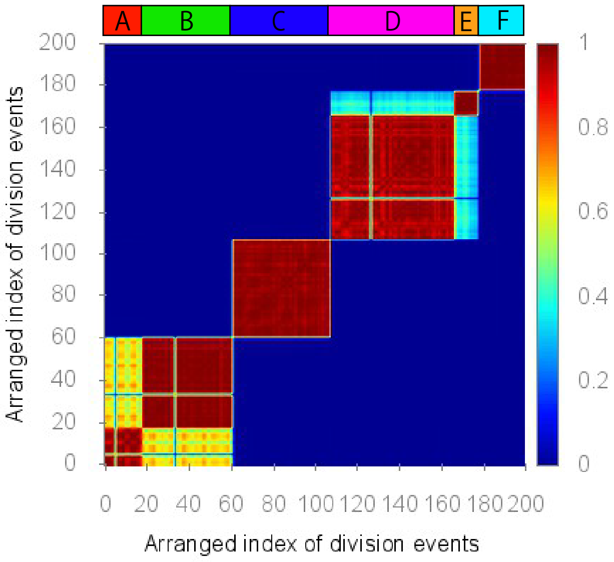

FIG. 8: The indices are arranged from Fig. 2(III)(iii) in the main text to categorize into each type $A - F$.
